# Green Synthesized of Ag/Ag_2_O Nanoparticles Using Aqueous Leaves Extracts of *Phoenix dactylifera* L. and Their Azo Dye Photodegradation

**DOI:** 10.3390/membranes11070468

**Published:** 2021-06-25

**Authors:** Salah Eddine Laouini, Abderrhmane Bouafia, Alexander V. Soldatov, Hamed Algarni, Mohammed Laid Tedjani, Gomaa A. M. Ali, Ahmed Barhoum

**Affiliations:** 1 Department of Process Engineering and Petrochemistry, Faculty of Technology, University of Echahid Hamma Lakhdar El Oued, El-Oued 39000, Algeria; salah_laouini@yahoo.fr (S.E.L.); medlaidtedjani@gmail.com (M.L.T.); 2 The Smart Materials Research Institute, Southern Federal University, Sladkova Str. 178/24, Rostov-on-Don 344090, Russia; soldatov@sfedu.ru; 3 Research Centre for Advanced Materials Science (RCAMS), King Khalid University, P.O. Box 9004, Abha 61413, Saudi Arabia; halgarni@kku.edu.sa; 4 Department of Physics, Faculty of Sciences, King Khalid University, P.O. Box 9004, Abha 61413, Saudi Arabia; 5 Chemistry Department, Faculty of Science, Al–Azhar University, Assiut 71524, Egypt; 6 NanoStruc Research Group, Chemistry Department, Faculty of Science, Helwan University, Helwan 11795, Egypt; 7 School of Chemical Sciences, Fraunhofer Project Centre, Dublin City University, D09 V209 Dublin, Ireland

**Keywords:** silver/silver oxide nanoparticles, *Phoenix dactylifera* L., photosynthesis, catalytic activity, dye degradation

## Abstract

In this study, silver/silver oxide nanoparticles (Ag/Ag_2_O NPs) were successfully biosynthesized using *Phoenix dactylifera* L. aqueous leaves extract. The effect of different plant extract/precursor contractions (volume ratio, *v*/*v*%) on Ag/Ag_2_O NP formation, their optical properties, and photocatalytic activity towards azo dye degradation, i.e., Congo red (CR) and methylene blue (MB), were investigated. X-ray diffraction confirmed the crystalline nature of Ag/Ag_2_O NPs with a crystallite size range from 28 to 39 nm. Scanning electron microscope images showed that the Ag/Ag_2_O NPs have an oval and spherical shape. UV–vis spectroscopy showed that Ag/Ag_2_O NPs have a direct bandgap of 2.07–2.86 eV and an indirect bandgap of 1.60–1.76 eV. Fourier transform infrared analysis suggests that the synthesized Ag/Ag_2_O NPs might be stabilized through the interactions of -OH and C=O groups in the carbohydrates, flavonoids, tannins, and phenolic acids present in *Phoenix dactylifera* L. Interestingly, the prepared Ag/Ag_2_O NPs showed high catalytic degradation activity for CR dye. The photocatalytic degradation of the azo dye was monitored spectrophotometrically in a wavelength range of 250–900 nm, and a high decolorization efficiency (84.50%) was obtained after 50 min of reaction. As a result, the use of *Phoenix dactylifera* L. aqueous leaves extract offers a cost-effective and eco-friendly method.

## 1. Introduction

Plant extract synthesis is a modern area of biotechnology that is economically and environmentally beneficial as an alternative to chemical and physical methods that contain part of the hazard to the environment [1,2]. It is a relatively new scientific field that adopts engineering nanoparticles ranging from metals, metal oxides, and hybrids [3,4,5]. This approach is biologically safe, non-toxic, and environmentally friendly, as natural plant extracts are typically used [6,7]. The biomolecules present in the plant extract can reduce metal ions to nanoparticles (NPs) in a single-step green synthesis process [6,8]. The plant-extract reduction of this metal ion to base metal is very rapid, is easy at room temperature and pressure, and is easily scaled up [9]. The synthesis mediated by plant extracts is environmentally friendly. The reducing agents involved include various water-soluble plant metabolites (alkaloids, phenolic compounds, terpenoids, etc.) and co-enzymes. Ag NPs have become a particular focus of plant-based synthesis. Extracts from various plant species are used to prepare Ag NPs [9].

Among metal oxides, silver oxide (AgO_x_) has received a great deal of attention as it may be used in several areas, including the synthesis of nanoscale electronics, improved surface Raman properties, and more [10]. Various accessible compositions of silver oxides include Ag_2_O, Ag_2_O_3_, AgO, and Ag_3_O_4_. Ag_2_O semiconductors exist in various forms, with a reported bandgap ranging from 1.2 eV to 3.4 eV [11]. Due to its photosensitivity and instability under irradiation, Ag_2_O not only functions as a co-catalyst but is also often used as a major photocatalytic material [12,13]. Ag_2_O NPs can be prepared by combining aqueous AgNO_3_ solutions and alkali hydroxide [14]. The Ag_2_O NPs are also used as a mild oxidizing agent in organic synthesis; they oxidize aldehydes to carboxylic acids. Recently, researchers have made a great effort to synthesize Ag and Ag_2_O NPs by various chemical reagents [15,16]. Various methods that can be used to synthesize Ag NPs include chemical reduction [17], electrochemistry [18], laser ablation [19], electron irradiation [20], gamma irradiation [21], photochemical methods [22], Langmuir–Blodgett [23], and synthetic biological methods [24]. However, these methods usually use either expensive or toxic chemicals (e.g., reducing agents and stabilizers). Additionally, the residual by-products may make Ag and Ag_2_O NPs unsuitable for biomedical applications, while differential responses of Ag and Ag_2_O NPs might be normal due to different synthesis methods and affectability of various cell types. Research has detailed the inhibition of cell practicality in the 25–50 μg/mL range for Ag NPs in an immediate correlation of normal human fibroblasts and glioblastoma cells [25].

Silver-based nanocatalysts, such as silver halides (AgCl, AgBr, AgI) [26], carbonates (Ag_2_CO_3_) [17], phosphates (Ag_3_PO_4_) [27], chromates (Ag_2_CrO_4_), oxides (Ag_2_O), sulfides (Ag_2_S) have shown the ability to decompose organic pollutants into final products (CO_2_, H_2_O, etc.) under visible light irradiation. Ag_2_O is regarded as a promising candidate due to its unique electronic structure, crystal, and band structure. Ag_2_O generally exhibits a narrow direct bandgap of 1.2–1.4 eV. Assuming a bandgap of about 1.4 eV, the edge positions of the valence band and conduction band are determined to be 0.09 and 1.49 eV, respectively [28]. Previously, under the induction of fluorescence, Ag_2_O has succeeded in decolorizing an aqueous solution of anionic dyes (methyl orange) at a rate constant of 0.023 min^−1^, demonstrating its photocatalytic efficiency [12]. Visible light-induced dye degradation has aroused great interest, and some research groups have reported the use of metal oxides to degrade azo dyes (e.g., methylene blue (MB)) effectively [29]. Comparative studies on the photocatalytic degradation of visible-light-induced MB using different metal oxide catalysts have shown their rate constants. Recently, a research team successfully synthesized Ag_2_O NPs using the green combustion method. These NPs were then used to evaluate the photocatalytic degradation of azo dye (Acid orange 8) under UV light irradiation with a degradation efficiency of 70% [30]. Shah et al. [31] examined the photocatalytic activity of the synthesized Ag_2_O NPs by applying an environmentally friendly solution (P.emodi’s fresh leaf extract). Within 180 min, the Ag_2_O NPs used UV-light to degrade 97.8% of MB dye with a rate constant of 0.0214 min^−1^ [31]. Ying and others used the manufactured Ag_2_O NPs for photocatalytic-assisted adsorption to remove azo dyes such as Congo red (CR). The results illustrate the regeneration of Ag_2_O NPs after the complete photodegradation of the CR dye within 35 min under visible light irradiation. According to the Langmuir equation, the dye adheres to pseudo-second-order kinetics [32].

Azo dyes are the most widely used dyes in the textile industry. However, the degree of azo dye’s exhaustion onto textile products is never complete [33]. About 15–20% of the total azo dye is discharged into the industry effluents, harming the environment [34]. It is worth mentioning herein that the biodegradation of azo dyes is difficult due to their complex structure and synthetic nature. Azo dyes are exceptionally toxic and conceivably carcinogenic [35,36] and may cause different infections in animals and people [36,37]. Therefore, azo dye’s removal from industry effluents is desirable for aesthetic reasons and because the azo dye products are highly toxic to aquatic life and mutagenic to humans [25]. 

This study investigates an efficient and sustainable route of Ag/Ag_2_O NP preparation from aqueous AgNO_3_ using *Phoenix dactylifera* L. aqueous leaves extract. The effect of different plant extract/precursor contractions (volume ratios % *v*/*v*) on Ag/Ag_2_O NP formation and their optical properties and catalytic activity towards CR and MB dyes degradation were investigated. The synthesized Ag/Ag_2_O NPs were characterized by ultraviolet–visible spectroscopy (UV–vis), scanning electron microscopy (SEM), and Fourier transform infrared spectroscopy (FTIR) to determine the properties of the bioactive ingredients (capping agent) in each leaf extract [38]. CR is a benzidine-based anionic diazo dye with two azo bonds (-N=N-) chromophores in the molecule [39]. CR and MB dyes were selected for this study because they are the most common azo dyes used in the textile industry. This class of dyes is known to be metabolized to human carcinogens and mutagens, benzidine. Therefore, its use is banned in many countries due to its structural stability; it has high toxicity [40]. This study evaluates the biodegradable and detoxifying effects of Ag/Ag_2_O NPs on CR and MB dye degradation under different reaction times. 

## 2. Results and Discussion

### 2.1. Crystal Structure and Composition

**Figure 1:**
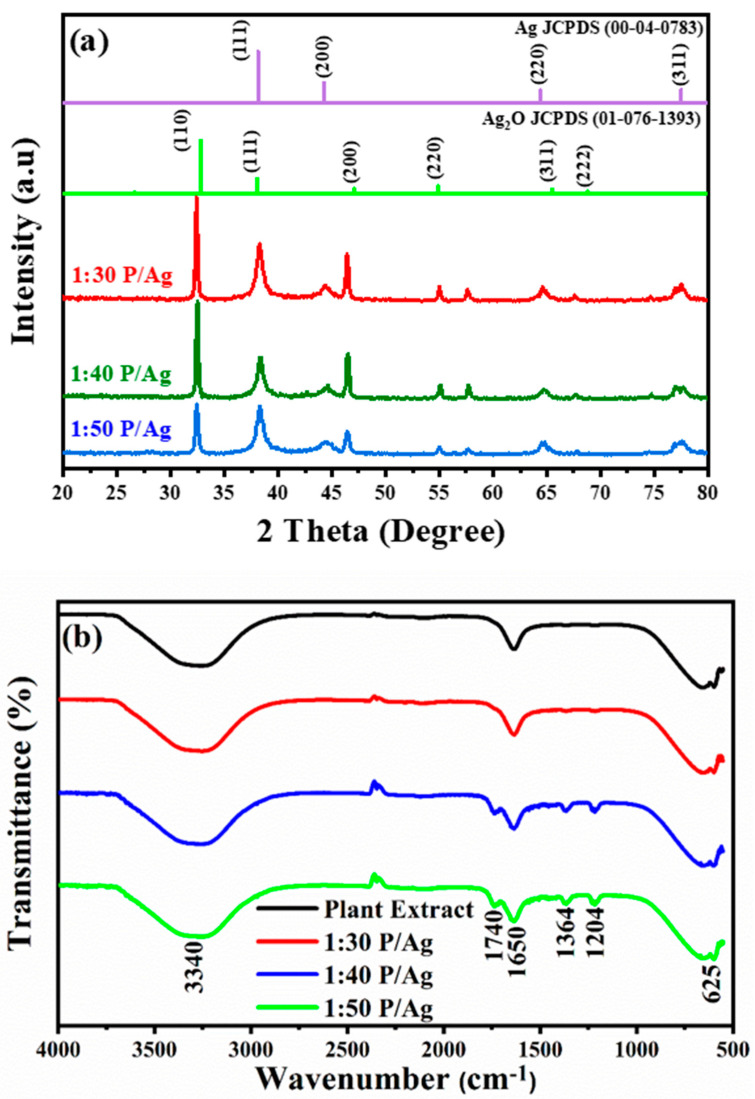
(**a**) XRD patterns; (**b**) FTIR spectra of Ag/Ag_2_O at different volume ratios of *Phoenix dactylifera* L. extract.

The effect of *Phoenix dactylifera* L. aqueous leaves extract on the obtained powder of Ag/Ag_2_O was investigated using various structural and morphological analyses. XRD analysis of the biosynthesized Ag/Ag_2_O NPs at different volume ratios (Ag ions solution:extract) is shown in Figure 1a. Bragg reflection peaks in all patterns located at 2θ values of 38.16°, 44.34°, 64.57°, and 77.60° correspond to (111), (200), (220), and (311) planes of metallic Ag based on the face-centered cubic structure (JCPDS, file No. 04-0783) [41,42], whereas the peaks at 2θ values of 26.90°, 32.69°, 37.94°, 54.90°, 65.54°, and 69.00° are related to (110), (111), (200), (220), (311) and (222) planes of Ag_2_O face-centered cubic crystalline (JCPDS, file No. 01-076-1393) [43]. The crystallite size of the synthesized NPs was estimated, selecting the peak of highest intensity situated at a 2θ value of 38.16° and 32.69° for Ag nanocrystals and Ag_2_O nanocrystals, respectively, using the Scherrer formula [44]. Table 1 shows that the crystallite size is affected by the volume ratio. It is noteworthy that increasing the volume ratio from 1:30 to 1:40 did not significantly affect the Ag/Ag_2_O NPs crystallite size. On the other hand, increasing the volume ratio from 1:40 to 1:50 decreased the crystallite size of Ag/Ag_2_O significantly from 37.71 nm to 28.66 nm. From Figure 1a, it can be noted that the higher the intensity of the peaks, the greater the size of the crystals, and this supports the idea that the more it decreases the volume ratio between plant extract/precursor silver contraction (*v*/*v*%), the greater the size of the crystals; the decrease in crystallite size by increasing the amount of surfactant (plant extract) ratio has also been reported previously [45].

FTIR analysis (Figure 1b) was carried out to identify the potential presence of reducing and stabilizing biomolecules in the *Phoenix dactylifera* L. extract on the surface of the Ag-Ag_2_O. The resultant FTIR spectra exhibited several absorption bands corresponding to the functional groups of the biomolecules existing in the plant extract. Five main absorption bands were observed: the broadband centered at 3340 cm^−1^ is assigned to O–H stretching vibrations [46], and the intense band at 1650 cm^−1^ is due to C=O stretching and N–H bending vibrations of the primary amides group, which is commonly found in the protein [47]. The absorption bands situated around 1740 and 1364 cm^−1^ correspond to the stretching vibrations of C=C, C–C, and C–O of the aromatics cycles [48]. The band 1204 cm^−1^ is assigned to the nitro banding N-O vibrations and C-O-C stretching vibrations of the aromatic ring [49]. Besides, the band located at 650 cm^−1^ corresponds to C–H bending vibrations out of the plane [50]. In addition, the Ag–O bond vibration band is observed at 625 cm^−1^ [51]. The FTIR results show that *Phoenix dactylifera* L. extract contains many different functional groups, such as carboxyl, carbonyls, amides, and phenols, serving as bioreducing and capping agents for Ag/Ag_2_O synthesis [52]. These groups have a critical role in interactions and binding between Ag and the extract molecules and stabilizing the final product [53].

### 2.2. Particle Size and Morphology 

**Figure 2:**
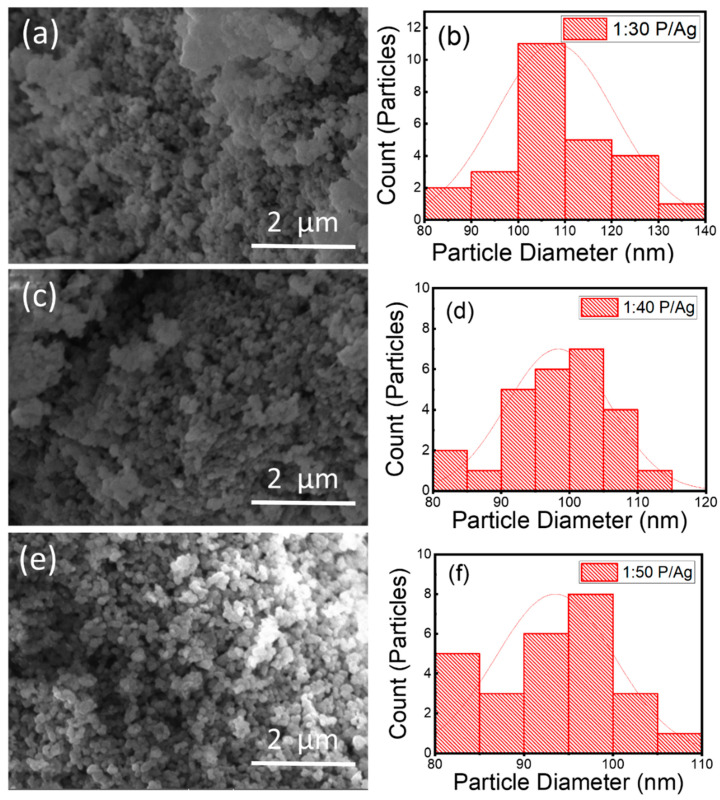
SEM images and particle size distributions of green synthesized Ag/Ag_2_O NPs with different volume ratios: (**a**,**b**) 1:30 P/Ag, (**c**,**d**) 1:40 P/Ag, (**e**,**f**) 1:50 P/Ag.

SEM was used to study the formation of Ag/Ag_2_O NPs and their morphological size. Figure 2 shows the SEM images of the synthesized Ag/Ag_2_O NPs with different volume ratios: 1:30 P/Ag, 1:40 P/Ag, and 1:50 P/Ag. The Ag/Ag_2_O NPs were oval and spherical in shape. A similar phenomenon has been reported in previous studies [54,55]. Most of the Ag/Ag_2_O NPs were aggregated, and a few individual particles were also observed. Finally, shown in Figure 2b,d,f is the average particle size distribution of Ag/Ag_2_O NPs, mainly around 100 nm. 

### 2.3. UV–Visible Spectroscopy and Bandgap

**Figure 3:**
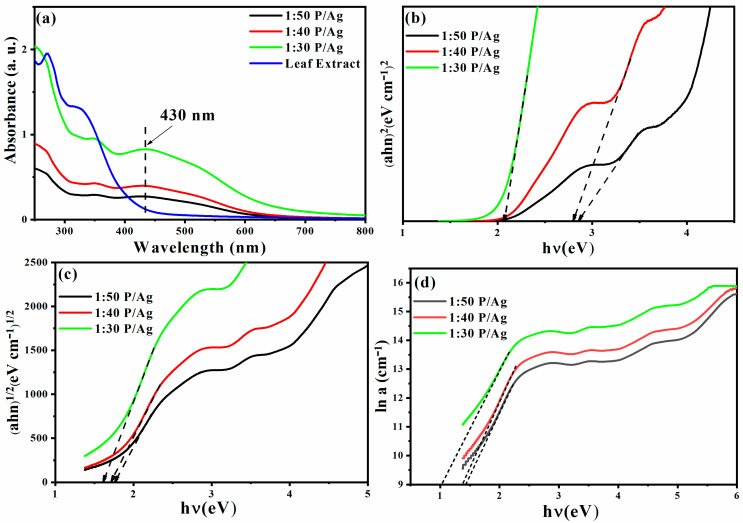
UV–visible spectra (**a**); determination of optical energy gap for direct (**b**) and indirect (**c**) transitions using Tauc’s method; calculation of Urbach energy (**d**) for Ag/Ag_2_O NPs.

When light strikes the Ag/Ag_2_O NPs, they are excited and exhibit a strong absorption band in the visible light region. This happens when the frequency of the electromagnetic field resonates with the motion of coherent electrons. This is called surface plasmon resonance (SPR) absorption. This feature makes UV–vis spectroscopy the most used method for determining the success of Ag/Ag_2_O NPs production. The colloidal solution of the as-prepared Ag/Ag_2_O and the plant extract were analyzed using UV–vis spectroscopy (Figure 3a). Accordingly, the plant extract spectrum exhibited two peaks at 275 and 320 nm. Meanwhile, the Ag/Ag_2_O spectra revealed a common peak in all the samples (1:30 P/Ag, 1:40 P/Ag, 1:50 P/Ag) situated at 430 nm. This peak corresponds to the characteristic surface plasmon resonance absorption band of Ag/Ag_2_O [56,57].

Reducing the amount of AgNO_3_ to *Phoenix dactylifera* L. extract shifts the SPR band to 275 nm and 320 nm, respectively. The blueshift of the SPR band is due to a size-dependent phenomenon called quantum confinement, the formation of smaller Ag/Ag_2_O NPs. Additionally, as the concentration (1:30 P/Ag, 1:40 P/Ag, 1:50 P/Ag) of the *Phoenix dactylifera* L. extract increases, the SPR band becomes narrower. The increase in strength may be due to an increase in the number of Ag/Ag_2_O NPs formed by the decrease in Ag^+^ ions in an aqueous solution. The increase in peak intensity may also be due to an increase in the number of Ag/Ag_2_O NPs formed by the decrease in Ag^+^ ions in an aqueous solution. In general, the optical bandgap of a semiconductor can be determined by plotting the relationship between the absorption coefficient and the photon energy. This can be estimated using Tauc’s formula (Equation (1)) [58,59]:

(1) $$\left(\alpha hv\right)=K{\left(hv-E_{g}\right)}^{n}$$

where *hυ* is the incident photon energy, *α* is the absorption coefficient, *K* is a constant, *E_g_* is the optical bandgap in electron volts (eV), and *n* is an exponent that can take different values depending on the nature of the electronic transition, i.e., *n* = 2 for direct transition, and *n* = 1/2 for indirect transition, as shown in Figure 3b,c [59,60,61,62]. The Urbach energy designates the width of the band tails of the localized states. The Urbach energy Eu, is determined from the slope of the linear part of the plot of lna versus photon energy (Figure 3d) [63]. The results of the estimated Urbach energy values of the samples are listed in Table 1.

(2) $$\ln a=\frac{hv}{E_{u}}+\;\mathrm{constant}\;(\ln a_{0})$$

To date, several methods have been reported for Ag NPs and Ag_2_O NP synthesis [64]. These have led to the technological development of relatively inexpensive Ag NPs and Ag_2_O NPs, but they require various chemicals that can affect the environment. Ag_2_O NPs can be synthesized by combining aqueous AgNO_3_ and alkali hydroxide (Ag^+^ + OH^−^ →AgOH). This reaction does not afford appreciable amounts of AgOH due to the favorable energetics for the following reaction: 2 AgOH → Ag_2_O + H_2_O (pK = 2.875). The synthesis of Ag_2_O NPs mediated by plant extracts is still unknown. The formation of Ag NPs and Ag_2_O NPs can also be mediated by polyphenols present in plant extracts. In this study, the green synthesis of Ag/Ag_2_O NPs was developed using aqueous leaves extracts of *Phoenix dactylifera* L. The phytochemical screening of *Phoenix dactylifera* L. leaves extract indicates the presence of polyphenols, flavonoids, and condensed tannins [65]. Total phenolics (2.78 ± 0.74 mg CAE/g plant extract), total flavonoids (22.84 ± 4.54 mg QE/mg plant extract), and condensed tannins (<1 mg CE/mg plant extract) [66,67,68] were calculated. The main role of the extract is to act as a reducing and capping agent to prevent the particles’ growth. These bioactive compounds contain hydroxyl (−OH) and ketonic (−C=O) groups that bind to the bulk metal Ag^+^ ion and reduce/cluster them to AgOH/Ag^0^ with a few nanometers size [69]. The concentration of Ag/Ag_2_O clusters in solution increased until it reached the supersaturation and, finally, reached the critical concentration of nucleation [19]. After that, the spontaneous nucleation of AgOH/Ag NPs occurred, and over time, many AgOH/Ag nuclei were formed, after which they grow into polydisperse spheres. As the reaction progresses, these spheres NPs grow to 80–120 nm size, where the hydroxyl (−OH) and ketonic (−C=O) groups present act as reducing agents and stabilizing agents in the synthesis of Ag/Ag_2_O NPs. AgOH is very unstable due to the high electronegativity of Ag^+^ and the different sizes of Ag^+^ and OH^−^ ions; it is readily oxidized to Ag_2_O upon drying. This may be the reason for the conversion existence of Ag_2_O in the prepared NPs instead of AgOH. In the existing literature, Ag_2_O NPs can be further reduced to Ag NPs [64], and in other experiments in our present work, we observed that the Ag_2_O phase is stable, and a complete reduction to metallic Ag was not observed. In our study, both Ag and Ag_2_O were detected. 

### 2.4. Photocatalytic Activity of Ag/Ag_2_O NPs for Azo Dye Degradation

**Figure 4:**
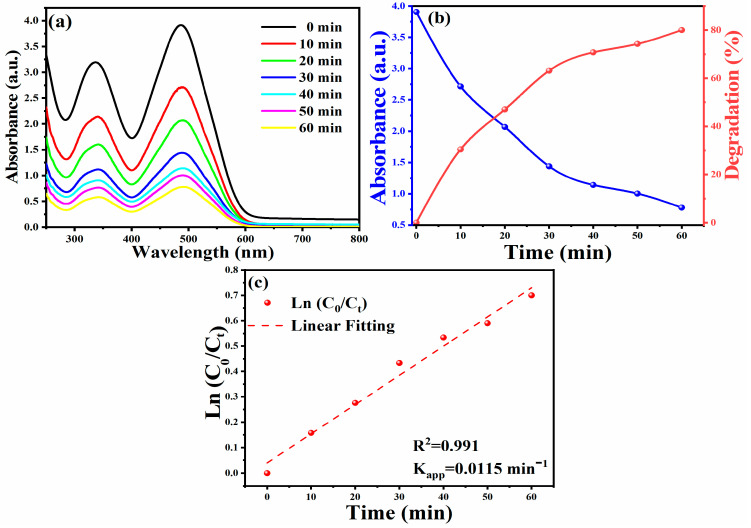
UV–vis spectra versus under optimal reaction conditions (**a**); absorbance and degradation versus time (**b**); plot of ln (*C*_0_/*C_t_*) versus time (**c**) for the Ag/Ag_2_O NPs catalyzed degradation of CR dye.

Upon applying optimal experimental conditions, an 80% degradation rate achieved within 60 min was observed in Figure 4a,b. The following formula calculates the degradation efficiency [70,71,72,73]:

$$\mathrm{Degradation}\;\mathrm{ratio}\;\left(\%\right)=\frac{C_{0}-C_{t}}{C_{t}}\times 100$$

where $C_{0}$ is the initial concentration of CR, and $C_{t}$ is the immediate concentration.

To determine the dye degradation kinetics of CR, the relationship between ln (*C*_0_/*C_t_*) and irradiation time was plotted (as shown in Figure 4c). It is found that under the catalysis of the silver/silver oxide nanocatalyst, the degradation reaction of CR obeys the first-order reaction kinetics [74,75]. Figure 4c shows a graph of ln (*C*_0_/*C_t_*) versus time, which helps one to understand the catalytic performance of biosynthetic Ag NPs. By plotting the relationship between ln (*C*_0_/*C_t_*) and time, the kinetic parameters of CR dye degradation under optimal reaction conditions were studied. As shown in Figure 4c, a linear relationship between ln (*C*_0_/*C_t_*) and time and the reaction follows pseudo-first-order kinetics. Therefore, the reaction rate is determined by *ln* (*C*_0_/*C_t_*) = *K_app_*. *T*, where *C*_0_ and *C_t_* are the concentration or absorbance of CR dye before and after degradation, K_app_ is the apparent rate (min^−1^). The apparent rate constant (k_app_) value is calculated from the straight-line slope using the above formula k_app_ = 0.01151 min^−1^ [76]. The results further prove that the Ag/Ag_2_O catalyst exhibits good photoreactivity, confirming the corresponding degradation efficiency [77].

**Figure 5:**
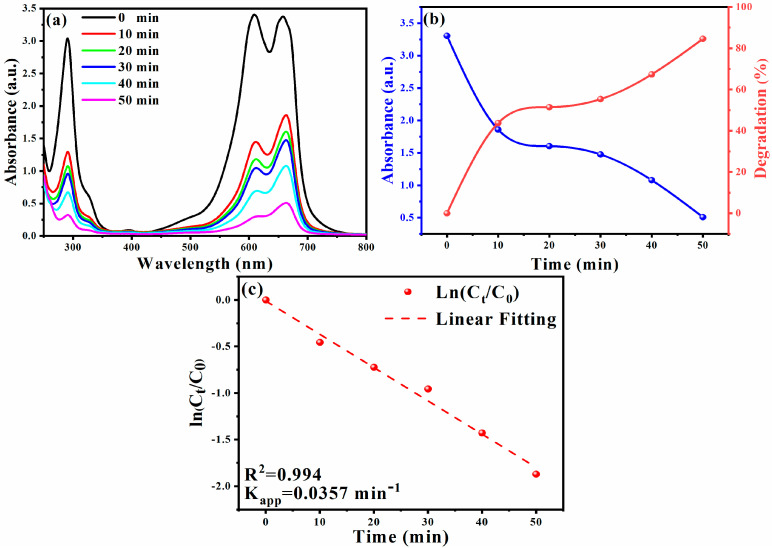
UV–vis spectra versus under optimal reaction conditions (**a**); absorbance and degradation versus time (**b**); the plot of ln (*C*_0_/*C_t_*) versus time (**c**) for the Ag/Ag_2_O NPs catalyzed degradation of MB dye.

The catalytic hydrolysis of the MB dye in the presence of sodium borohydride (NaBH_4_) was examined, which is another typical reaction to confirm the catalytic activity of Ag/Ag_2_O NPs and MB. The stimulation was monitored by UV–vis spectroscopy (Figure 5a). By adding Ag/Ag_2_O NPs as compounds to the reaction mixture, the catalytic reduction of the dye took place immediately. The strong blue color of the MB solution faded and became colorless after 8 min during the degradation process. The initial absorption peak at 663 nm gradually decreased over time, confirming the catalytic activity of the composite Ag/Ag_2_O NPs. The calculated degradation percentage as a quantitative expression of degraded dyes is shown in Figure 5b. The presence of amide groups of Ag/Ag_2_O NPs in transferring electrons from BH_4_^−^ anions to MB cations increased with increasing time, similar to the previously reported micro-Ag/Ag_2_O NPs [78,79,80,81,82,83,84].

The addition of biosynthetic Ag/Ag_2_O NPs improves the reduction process (the dye degrades up to 84.50% in 50 min). The analysis of the degradation reaction kinetics data showed pseudo-first-order reaction kinetics. The reaction rate is determined by ln (*C_t_/C*_0_) = −K_app_.t, where C_0_ and C_t_ are the concentration or absorbance of MB dye before and after degradation. The slope of the curve determines the k_app_ (min^−1^) value. The linear graph of ln (*C_t_/C*_0_) versus time (Figure 5c) supports the kinetic theory, where the k value is 0.137 min^−1^ [85]. 

The photocatalytic execution was evaluated by the bandgap, the oxidation capability of photograph-created openings, and the partition viability of photogenerated electrons and openings. The electronic structure and optical properties of Ag_2_O NPs were studied by hybrid density functional theory, reported earlier [86,87]. Bandgaps of the as-prepared Ag/Ag_2_O NPs were around 2.07–2.86 eV, which were enough for photocatalytic degradation of organic pollutants. The conduction bands of Ag_2_O NPs were mainly attributed to Ag 5s and 5p states, while the valence bands were dominated by O2p and Ag 4d states. Past reports demonstrated that Ag_2_O is flimsy under visible-light illumination and deteriorates into metallic Ag during the photocatalytic decay of natural substances.

**Figure 6:**
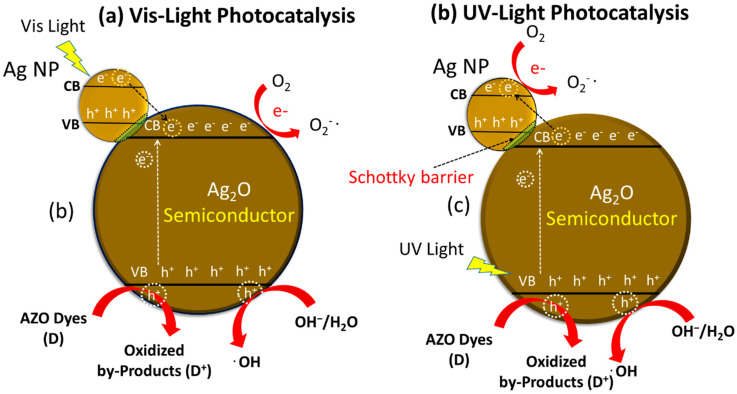
Schematic illustration of Ag/Ag_2_O towards photocatalytic degradation of organic pollutants (azo dye) under (**a**) visible light irradiation, (**b**) UV light irradiation.

Notwithstanding, after the fractional in situ development of Ag on the outside of Ag_2_O, the Ag/Ag_2_O NPs can function as a steady and proficient visible-light photocatalyst (Figure 6; CB: conduction band, VB: valence band, e^−^: electrons, h: holes) [12]. In view of this assumption, we endeavored to research the photocatalytic execution of Ag/Ag_2_O under fluorescent-light and visible-light irradiation in this examination. Besides, the stability and rehashed photocatalytic effectiveness of the Ag_2_O photocatalyst were examined. Under UV light, Ag NPs outside the Ag_2_O NPs upgraded the exchange of photogenerated electrons, delaying the charge carriers’ lifetime (Figure 6a). 

The Ag NPs framed on the outside of Ag_2_O NPs brought about the photo-activation of the catalyst in the visible range. The Ag_2_O NPs can gather noticeable light because of the small bandgap. Under the visible light irradiation, a couple of electrons (e^−^) in the conduction band (CB) and a hole (H^+^) in the valence band (VB) could be made on Ag NPs because of the surface Plasmon reverberation. Under visible light, the valance electron was moved from plasmonically energized Ag NPs to the conduction band of Ag_2_O NPs, the supposed Schottky intersection impact (Figure 6b).

CR comprises two phenyl rings attached to two naphthalene terminal pads containing amino and sulfonic gatherings. CR dye is a harmful and cancer-causing metabolite; CR dye is utilized in enterprises such as paper and elastic ventures and causes malignant bladder growth in people. Thus, its decrease is a significant issue because of its high natural harmfulness. The synergist debasement of CR was observed by biosynthetic Ag/Ag_2_O under different trial conditions. The CR fluid arrangement shows two tops at 340 nm and 490 nm in the UV–vis locale, which ties to the azo (-N=N-) bond. During the CR decrease measure, azo bonds in the color particle break down and produce different fragrant amine items (*sodium 4-amino-1-naphthalene sulfonate and 1,1′-Biphenyl*). The CR dye molecules cannot be diminished in the fluid medium within the presence of NaBH_4_ as the lessening specialist since this response is thermodynamically reachable yet is not dynamically conceivable. NaBH_4_ acts as an electron donor to Ag/Ag_2_O NPs. The NPs are the electron carrier from BH_4_^−^ (the donor) to the dye (the acceptor). BH_4_^−^ ions are nucleophilic, while the dye is electrophilic in nature concerning NPs, where the Ag NPs accept electrons from BH_4_^−^ ions and transferring them to the dye [88]. Consequently, the utilization of Ag/Ag_2_O NPs as nanocatalysts offers help and a pathway through the exchange of electrons between the beneficiary (CR dye) and the giver (borohydride particle, BH_4_^−^). Furthermore, the Ag/Ag_2_O NPs give an appropriate surface to restricting CR particles and borohydride particles (BH_4_^−1^) to associate with one another to shape decay items. A conceivable disintegration system for CR outside Ag/Ag_2_O NPs is introduced in Figure 6 [74,84]. 

## 3. Materials and Methods

### 3.1. Materials and Reagents 

Silver nitrate (AgNO_3_, 98%, VTRS Laboratory), distilled water, and the leaves of *Phoenix dactylifera* L. (collected from local fields in El Oued, Southeast of Algeria in autumn 2020) were used to synthesize Ag/Ag_2_O NPs. CR and MB dyes were supplied from *LOBA CHEMIE*, India. In addition, Merck supplied sodium borohydride 90% (NaBH_4_). 

### 3.2. Preparation of the Plant Extract 

The leaves of *Phoenix dactylifera* L. were washed by distilled water, dried for 12 days in a shaded place at room temperature, and then crushed to obtain a fine powder. An amount of 10 g of powdered *Phoenix dactylifera* L. leaves were added to 100 mL of distilled water into a 250 mL glass beaker to prepare the extract [8,66,89,90]. The mixture was stirred stably at room temperature for 24 h. After that, the extract was filtered with filter paper (Whatman No: 42) and stored in a glass container at 4 °C for further use (maximum storage was 1 month in dark conditions).

### 3.3. Biosynthesis of Ag/Ag_2_O Nanoparticles

Ag/Ag_2_O NPs were synthesized by adding different volume ratios (*v*/*v* %) of plant extract to silver nitrate solution (1 mM AgNO_3_) (1:30, 1:40, and 1:50). Briefly, 1 mL of leaf extract was added to 30, 40, or 50 mL of 1 mM AgNO_3_ aqueous solution in a 250 mL Erlenmeyer flask, stirred at 150 rpm at room temperature for 2 h, and the bioreduction of Ag^+^ to Ag was confirmed by the color change to brown after 5 min. The solid product was centrifuged at 3000 rpm for 15 min, and the remaining Ag/Ag_2_O NPs were washed with distilled water and dried in an oven at 100 °C for 24 h [91]. The dry powder was annealed at 500 °C for 3 h in the air atmosphere to fully crystallize the Ag/Ag_2_O NPs and remove the remaining organic compounds from the plant extract [92,93].

### 3.4. Characterization of Ag/Ag_2_O Nanoparticles

The crystalline structure of the synthesized NPs was obtained using an X-ray diffractometer (XRD, Rigaka Miniflex 600) and Cu-K_α_ radiation with a wavelength of 0.15406 nm in 2θ range 10–80°. The shape and morphology of the synthesis Ag/Ag_2_O NPs were confirmed by scanning electron microscope (SEM-TESCAN VEGA 3) at an accelerating voltage of 10 kV. Fourier transform infrared (FTIR) measurements of leaf extract and green synthesis Ag/Ag_2_O NPs were performed by a Nicolet iS5 (Thermo Fisher Scientific) to identify the functional groups carried out a range of 4000 to 400 cm^−1^. The optical characteristics of Ag/Ag_2_O NPs were analyzed using a UV–vis spectrophotometer (Shimadzu −1800). The measurement was recorded at the temperature in the wavelength region of 300 to 900 nm. The stability of Ag/Ag_2_O NPs was followed by UV–vis spectrometric measurements using a quartz cell and distilled water as a blank solution. 

### 3.5. Photocatalytic Degradation of Congo Red and Methylene Blue 

The catalytic degradation of dyes is a heterogeneous catalytic reaction with reactants in the aqueous phase (Azo dye in solution) and solid phase catalyst (Ag/Ag_2_O NPs). Thus, these surface-catalyzed reactions occur by a reaction between azo dye molecules adsorbed at the catalyst’s surface.

Ag/Ag_2_O NPs were used for the catalytic degradation of CR in the presence of sodium borohydride (NaBH_4_) at room temperature. The sample (1:50 P/Ag) was chosen for further photodegradation studies, as it showed the best structural and morphological characteristics based on the techniques used. Firstly, 2.5 mL of diluted CR was analyzed by UV–vis spectroscopy and appeared the peak occur at λ_max_ = 488 nm. The catalytic reaction was calculated to be conducted in all experiments. To investigate the catalytic effect of the Ag/Ag_2_O NPs, the NaBH_4_ solution (10^−2^ M) (considered a reducing agent) was added to the CR solution (10^−4^ M), which was followed by the addition of the Ag/Ag_2_O NPs (10 mg/L). The pH of the reaction was adjusted and the reaction was completed at the volume of 5 mL. The degradation process was observed spectrophotometrically in a wavelength range of 250–900 nm at 10–60 min. The decolorization process was observed as a decline in the absorbance intensity (λ_max_) of the solution. The experiments examined the catalytic efficacy of the Ag/Ag_2_O NPs on suspension. 

The MB dye degradation reaction composed 3 mL of MB solution (2 × 10^−6^ M, pH = 6.4). The above reaction was added to 2.25 mL of NaBH_4_ solution (6 × 10^−6^ M) and 150 µL (80.85 mg/L Ag/Ag_2_O NPs on suspension). The degradation of MB was controlled at different times by optical absorbance at 611 and 663 nm. 

## 4. Conclusions

Plant extract-mediated and green biomimetic synthesis can be considered green technology for the rapid production of Ag/Ag_2_O NPs. This method successfully meets the excessive current market demand of many types of nanoparticles, reducing the employment or production of substances harmful to human health and the environment. In this study, the green synthesis of Ag/Ag_2_O NPs was successfully performed using *Phoenix dactylifera* L. aqueous leaf extract. The process is relatively easy, fast, cheap, environmentally friendly, and does not require any organic solvents or other toxic reagents. Therefore, this synthesis method is more beneficial than conventional methods for synthesizing Ag/Ag_2_O NPs. The shape of the prepared Ag/Ag_2_O NPs is close to the spherical crystal in nature, with an average crystallite size of 28–39 nm. Besides, this study shows that the prepared Ag/Ag_2_O NPs have excellent photocatalytic activity for azo dye degradation. The photocatalytic degradation of the methylene blue and Congo red was a high decolorization efficiency (84.5%) was obtained after 50 min of reaction. The prepared photocatalyst Ag/Ag_2_O NPs help treat wastewater (dye degradation) in medicines, cosmetics, paints, plastics, and textiles.

## Figures and Tables

**Table 1:** The effect of volume ratio on the characteristics of the prepared Ag/Ag_2_O NPs.

Samples	Crystallite Size (nm)	Direct Optical Bandgap (Ev)	Indirect Optical Bandgap (Ev)	Urbach Energy (Ev)
1:30 P/Ag	39.40 ± 1.45	2.07	1.60	0.22
1:40 P/Ag	37.71 ± 0.61	2.80	1.70	0.20
1:50 P/Ag	28.66 ± 1.12	2.86	1.76	0.23

## References

[1] Barhoum A., Jeevanandam J., Rastogi A., Samyn P., Boluk Y., Dufresne A., Danquah M.K., Bechelany M. (2020). Plant celluloses, hemicelluloses, lignins, and volatile oils for the synthesis of nanoparticles and nanostructured materials. Nanoscale.

[2] Ali G.A.M., Barhoum A., Gupta V.K., Nada A.A., El-Maghrabi H.H., Kanthasamy R., Shaaban E.R., Algarni H., Chong K.F. (2020). High surface area mesoporous silica for hydrogen sulfide effective removal. Curr. Nanosci..

[3] Khare S., Williams K., Gokulan K., Batt C.A., Tortorello M.L. (2014). Nanotechnology.

[4] Abdel Ghafar H.H., Ali G.A.M., Fouad O.A., Makhlouf S.A. (2015). Enhancement of adsorption efficiency of methylene blue on Co_3_O_4_/SiO_2_ nanocomposite. Desalin. Water Treat..

[5] Sadegh H., Ali G.A.M., Makhlouf A.S.H., Chong K.F., Alharbi N.S., Agarwal S., Gupta V.K. (2018). MWCNTs-Fe_3_O_4_ nanocomposite for Hg(II) high adsorption efficiency. J. Mol. Liq..

[6] Rastogi A., Singh P., Haraz F.A., Barhoum A., Barhoum A., Makhlouf A.S.H. (2018). Biological Synthesis of Nanoparticles: An Environmentally Benign Approach.

[7] Wibowo A., Tajalla G.U.N., Marsudi M.A., Cooper G., Asri L.A.T.W., Liu F., Ardy H., Bartolo P.J.D.S. (2021). Green Synthesis of Silver Nanoparticles Using Extract of Cilembu Sweet Potatoes (Ipomoea batatas L var. Rancing) as Potential Filler for 3D Printed Electroactive and Anti-Infection Scaffolds. Molecules.

[8] Tahir K., Nazir S., Ahmad A., Li B., Ali Shah S.A., Khan A.U., Khan G.M., Khan Q.U., Haq Khan Z.U., Khan F.U. (2016). Biodirected synthesis of palladium nanoparticles using Phoenix dactylifera leaves extract and their size dependent biomedical and catalytic applications. RSC Adv..

[9] Said M.M., Rehan M., El-Sheikh S.M., Zahran M.K., Abdel-Aziz M.S., Bechelany M., Barhoum A. (2021). Multifunctional hydroxyapatite/silver nanoparticles/cotton gauze for antimicrobial and biomedical applications. Nanomaterials.

[10] Kiani F.A., Shamraiz U., Badshah A. (2020). Enhanced photo catalytic activity of Ag_2_O nanostructures through strontium doping. Mater. Res. Express.

[11] Reddy P.N., Reddy M.H.P., Pierson J.F., Uthanna S. (2014). Characterization of Silver Oxide Films Formed by Reactive RF Sputtering at Different Substrate Temperatures. ISRN Opt..

[12] Wang X., Li S., Yu H., Yu J., Liu S. (2011). Ag_2_O as a New Visible-Light Photocatalyst: Self-Stability and High Photocatalytic Activity. Chem. Eur. J..

[13] Zhang M., Du H., Ji J., Li F., Lin Y.C., Qin C., Zhang Z., Shen Y. (2021). Highly Efficient Ag_3_PO_4_/g-C_3_N_4_ Z-Scheme Photocatalyst for Its Enhanced Photocatalytic Performance in Degradation of Rhodamine B and Phenol. Molecules.

[14] Barhoum A., Rahier H., Benelmekki M., Assche G.V., Barhoum A., Makhlouf A.S.H. (2018). Recent Trends in Nanostructured Particles: Synthesis, Functionalization, and Applications.

[15] Liu M., Wang H., Zeng H., Li C.-J. (2015). Silver(I) as a widely applicable, homogeneous catalyst for aerobic oxidation of aldehydes toward carboxylic acids in water—“silver mirror”: From stoichiometric to catalytic. Sci. Adv..

[16] Rehan M., Barhoum A., Van Assche G., Dufresne A., Gätjen L., Wilken R. (2017). Towards multifunctional cellulosic fabric: UV photo-reduction and in-situ synthesis of silver nanoparticles into cellulose fabrics. Int. J. Biol. Macromol..

[17] Barhoum A., Rehan M., Rahier H., Bechelany M., Van Assche G. (2016). Seed-Mediated Hot-Injection Synthesis of Tiny Ag Nanocrystals on Nanoscale Solid Supports and Reaction Mechanism. ACS Appl. Mater. Interfaces.

[18] Sun Y., Yin Y., Mayers B.T., Herricks T., Xia Y. (2002). Uniform Silver Nanowires Synthesis by Reducing AgNO_3_ with Ethylene Glycol in the Presence of Seeds and Poly(Vinyl Pyrrolidone). Chem. Mater..

[19] Karatutlu A., Barhoum A., Sapelkin A. (2018). Liquid-phase synthesis of nanoparticles and nanostructured materials. Emerging Applications of Nanoparticles and Architecture Nanostructures.

[20] Prasad S., Kumar V., Kirubanandam S., Barhoum A. (2018). Engineered Nanomaterials: Nanofabrication and Surface Functionalization.

[21] Dimitrijevic N.M., Bartels D.M., Jonah C.D., Takahashi K., Rajh T. (2001). Radiolytically Induced Formation and Optical Absorption Spectra of Colloidal Silver Nanoparticles in Supercritical Ethane. J. Phys. Chem. B.

[22] Callegari A., Tonti D., Chergui M. (2003). Photochemically Grown Silver Nanoparticles with Wavelength-Controlled Size and Shape. Nano Lett..

[23] Zhang L., Shen Y., Xie A., Li S., Jin B., Zhang Q. (2006). One-Step Synthesis of Monodisperse Silver Nanoparticles beneath Vitamin E Langmuir Monolayers. J. Phys. Chem. B.

[24] Naik R.R., Stringer S.J., Agarwal G., Jones S.E., Stone M.O. (2002). Biomimetic synthesis and patterning of silver nanoparticles. Nat. Mater..

[25] Karekar N., Karan A., Khezerlou E., Prajapati N., Pernici C.D., Murray T.A., DeCoster M.A. (2019). Self-Assembled Metal—Organic Biohybrids (MOBs) Using Copper and Silver for Cell Studies. Nanomaterials.

[26] Rehan M., Khattab T.A., Barohum A., Gätjen L., Wilken R. (2018). Development of Ag/Ag_X_ (X = Cl, I) nanoparticles toward antimicrobial, UV-protected and self-cleanable viscose fibers. Carbohydr. Polym..

[27] Rehan M., Barhoum A., Khattab T.A., Gätjen L., Wilken R. (2019). Colored, photocatalytic, antimicrobial and UV-protected viscose fibers decorated with Ag/Ag_2_CO_3_ and Ag/Ag_3_PO_4_ nanoparticles. Cellulose.

[28] Kumar S., Verma A., Pal S., Sinha I. (2018). Curcumin-Functionalized Ag/Ag_2_O Nanocomposites: Efficient Visible-Light Z-scheme Photocatalysts. Photochem. Photobiol..

[29] Yang Z.-H., Ho C.-H., Lee S. (2015). Plasma-induced formation of flower-like Ag_2_O nanostructures. Appl. Surf. Sci..

[30] Rashmi B.N., Harlapur S.F., Avinash B., Ravikumar C.R., Nagaswarupa H.P., Anil Kumar M.R., Gurushantha K., Santosh M.S. (2020). Facile green synthesis of silver oxide nanoparticles and their electrochemical, photocatalytic and biological studies. Inorg. Chem. Commun..

[31] Shah A., Haq S., Rehman W., Waseem M., Shoukat S., Rehman M.-U. (2019). Photocatalytic and antibacterial activities of paeonia emodi mediated silver oxide nanoparticles. Mater. Res. Express.

[32] Wang Y., Bi N., Zhang H., Tian W., Zhang T., Wu P., Jiang W. (2020). Visible-light-driven photocatalysis-assisted adsorption of azo dyes using Ag_2_O. Colloids Surf. A Physicochem. Eng. Asp..

[33] Koe W.S., Lee J.W., Chong W.C., Pang Y.L., Sim L.C. (2020). An overview of photocatalytic degradation: Photocatalysts, mechanisms, and development of photocatalytic membrane. Environ. Sci. Pollut. Res..

[34] Donkadokula N.Y., Kola A.K., Naz I., Saroj D. (2020). A review on advanced physico-chemical and biological textile dye wastewater treatment techniques. Rev. Environ. Sci. BioTechnol..

[35] Sharma B., Dangi A.K., Shukla P. (2018). Contemporary enzyme based technologies for bioremediation: A review. J. Environ. Manag..

[36] Lellis B., Fávaro-Polonio C.Z., Pamphile J.A., Polonio J.C. (2019). Effects of textile dyes on health and the environment and bioremediation potential of living organisms. Biotechnol. Res. Innov..

[37] Khan S., Malik A. (2018). Toxicity evaluation of textile effluents and role of native soil bacterium in biodegradation of a textile dye. Environ. Sci. Pollut. Res..

[38] Barhoum A., Luisa García-Betancourt M. (2018). Physicochemical Characterization of Nanomaterials: Size, Morphology, Optical, Magnetic, and Electrical Properties.

[39] Liu X., Li W., Chen N., Xing X., Dong C., Wang Y. (2015). Ag–ZnO heterostructure nanoparticles with plasmon-enhanced catalytic degradation for Congo red under visible light. RSC Adv..

[40] Özcan A., Oturan M.A., Oturan N., Şahin Y. (2009). Removal of Acid Orange 7 from water by electrochemically generated Fenton’s reagent. J. Hazard. Mater..

[41] Priyadharshini R.I., Prasannaraj G., Geetha N., Venkatachalam P. (2014). Microwave-Mediated Extracellular Synthesis of Metallic Silver and Zinc Oxide Nanoparticles Using Macro-Algae (Gracilaria edulis) Extracts and Its Anticancer Activity Against Human PC3 Cell Lines. Appl. Biochem. Biotechnol..

[42] Meng Y. (2015). A sustainable approach to fabricating ag nanoparticles/PVA hybrid nanofiber and its catalytic activity. Nanomaterials.

[43] Rajabi A., Ghazali M.J., Mahmoudi E., Azizkhani S., Sulaiman N.H., Mohammad A.W., Mustafah N.M., Ohnmar H., Naicker A.S. (2018). Development and antibacterial application of nanocomposites: Effects of molar ratio on Ag_2_O–CuO nanocomposite synthesised via the microwave-assisted route. Ceram. Int..

[44] Patterson A.L. (1939). The Scherrer Formula for X-Ray Particle Size Determination. Phys. Rev..

[45] Moya C., Batlle X., Labarta A. (2015). The effect of oleic acid on the synthesis of Fe_3-x_O_4_ nanoparticles over a wide size range. Phys. Chem. Chem. Phys..

[46] Bouafia A., Laouini S.E., Khelef A., Tedjani M.L., Guemari F. (2020). Effect of Ferric Chloride Concentration on the Type of Magnetite (Fe_3_O_4_) Nanoparticles Biosynthesized by Aqueous Leaves Extract of Artemisia and Assessment of Their Antioxidant Activities. J. Clust. Sci..

[47] Auti A.M., Narwade N.P., Deshpande N.M., Dhotre D.P. (2019). Microbiome and imputed metagenome study of crude and refined petroleum-oil-contaminated soils: Potential for hydrocarbon degradation and plant-growth promotion. J. Biosci..

[48] Ismail M., Khan M.I., Akhtar K., Khan M.A., Asiri A.M., Khan S.B. (2018). Biosynthesis of silver nanoparticles: A colorimetric optical sensor for detection of hexavalent chromium and ammonia in aqueous solution. Phys. E.

[49] Raj A., Lawrence R., Lawrence K., Silas N., Jaless M., Srivastava R. (2018). Green synthesis and charcterization of silver nanoparticles from leafs extracts of rosa indica and its antibacterial activity against human pathogen bacteria. Orient. J. Chem..

[50] Arif D., Niazi M.B.K., Ul-Haq N., Anwar M.N., Hashmi E. (2015). Preparation of antibacterial cotton fabric using chitosan-silver nanoparticles. Fibers Polym..

[51] Shoeb M., Mobin M., Ahmad S., Naqvi A.H. (2021). Facile synthesis of polypyrrole coated graphene Gr/Ag–Ag_2_O/PPy nanocomposites for a rapid and selective response towards ammonia sensing at room temperature. J. Sci. Adv. Mater. Devices.

[52] Laid T.M., Abdelhamid K., Eddine L.S., Abderrhmane B. (2020). Optimizing the biosynthesis parameters of iron oxide nanoparticles using central composite design. J. Mol. Struct..

[53] Nanaei M., Nasseri M.A., Allahresani A., Kazemnejadi M. (2019). Phoenix dactylifera L. extract: Antioxidant activity and its application for green biosynthesis of Ag nanoparticles as a recyclable nanocatalyst for 4-nitrophenol reduction. SN Appl. Sci..

[54] Stoehr L.C., Gonzalez E., Stampfl A., Casals E., Duschl A., Puntes V., Oostingh G.J. (2011). Shape matters: Effects of silver nanospheres and wires on human alveolar epithelial cells. Part. Fibre Toxicol..

[55] Elemike E.E., Onwudiwe D.C., Ekennia A.C., Sonde C.U., Ehiri R.C. (2017). Green Synthesis of Ag/Ag_2_O Nanoparticles Using Aqueous Leaf Extract of Eupatorium odoratum and Its Antimicrobial and Mosquito Larvicidal Activities. Molecules.

[56] Singh R., Wagh P., Wadhwani S., Gaidhani S., Kumbhar A., Bellare J., Chopade B.A. (2013). Synthesis, optimization, and characterization of silver nanoparticles from Acinetobacter calcoaceticus and their enhanced antibacterial activity when combined with antibiotics. Int. J. Nanomed..

[57] Karunakaran G., Jagathambal M., Gusev A., Kolesnikov E., Mandal A.R., Kuznetsov D. (2016). Allamanda cathartica flower’s aqueous extract-mediated green synthesis of silver nanoparticles with excellent antioxidant and antibacterial potential for biomedical application. MRS Commun..

[58] Strehlow W.H., Cook E.L. (1973). Compilation of Energy Band Gaps in Elemental and Binary Compound Semiconductors and Insulators. J. Phys. Chem. Ref. Data.

[59] Ali G.A.M., Fouad O.A., Makhlouf S.A. (2013). Structural, optical and electrical properties of sol-gel prepared mesoporous Co_3_O_4_/SiO_2_ nanocomposites. J. Alloys Compd..

[60] Mallick P., Dash B.N. (2013). X-ray diffraction and UV-visible characterizations of α-Fe_2_O_3_ nanoparticles annealed at different temperature. J. Nanosci. Nanotechnol..

[61] Jayaprakash P., Mohamed M.P., Caroline M.L. (2017). Growth, spectral and optical characterization of a novel nonlinear optical organic material: d-Alanine dl-Mandelic acid single crystal. J. Mol. Struct..

[62] Ali G.A.M., Fouad O.A., Makhlouf S.A., Yusoff M.M., Chong K.F. (2016). Optical and Electrochemical Properties of Co_3_O_4_/SiO_2_ Nanocomposite. Adv. Mater. Res..

[63] Martienssen W. (1957). Über die excitonenbanden der alkalihalogenidkristalle. J. Phys. Chem. Solids.

[64] Ravichandran S., Paluri V., Kumar G., Loganathan K., Kokati Venkata B.R. (2016). A novel approach for the biosynthesis of silver oxide nanoparticles using aqueous leaf extract of Callistemon lanceolatus (Myrtaceae) and their therapeutic potential. J. Exp. Nanosci..

[65] Boulenouar N., Marouf A., Cheriti A. (2011). Antifungal activity and phytochemical screening of extracts from Phoenix dactylifera L. cultivars. Nat. Prod. Res..

[66] Kriaa W., Fetoui H., Makni M., Zeghal N., Drira N.-E. (2012). Phenolic Contents and Antioxidant Activities of Date Palm (Phoenix dactylifera L.) Leaves. Int. J. Food Prop..

[67] Abuelgassim A.O. (2020). Towards the Utilization of Date Palm (Phoenix dactylifera) Leaves as a Rich Source of Antioxidants. J. Food Nutr. Res..

[68] John J.A., Shahidi F. (2019). Phenolic content, antioxidant and anti-inflammatory activities of seeds and leaves of date palm (Phoenix dactylifera L.). J. Food Bioact..

[69] Karatutlu A., Barhoum A., Sapelkin A. (2018). Theories of nanoparticle and nanostructure formation in liquid phase. Emerging Applications of Nanoparticles and Architecture Nanostructures.

[70] Ethiraj A.S., Uttam P., Varunkumar K., Chong K.F., Ali G.A.M. (2020). Photocatalytic performance of a novel semiconductor nanocatalyst: Copper doped nickel oxide for phenol degradation. Mater. Chem. Phys..

[71] Sharifi A., Montazerghaem L., Naeimi A., Abhari A.R., Vafaee M., Ali G.A.M., Sadegh H. (2019). Investigation of photocatalytic behavior of modified ZnS:Mn/MWCNTs nanocomposite for organic pollutants effective photodegradation. J. Environ. Manag..

[72] Giahi M., Pathania D., Agarwal S., Ali G.A.M., Chong K.F., Gupta V.K. (2019). Preparation of Mg-doped TiO_2_ nanoparticles for photocatalytic degradation of some organic pollutants. Studia Univ. Babes-Bolyai Chem..

[73] Pang Y.L., Law Z.X., Lim S., Chan Y.Y., Shuit S.H., Chong W.C., Lai C.W. (2021). Enhanced photocatalytic degradation of methyl orange by coconut shell–derived biochar composites under visible LED light irradiation. Environ. Sci. Pollut. Res..

[74] Royji Albeladi S.S., Malik M.A., Al-thabaiti S.A. (2020). Facile biofabrication of silver nanoparticles using Salvia officinalis leaf extract and its catalytic activity towards Congo red dye degradation. J. Mater. Res. Technol..

[75] Seyed Arabi S.M., Lalehloo R.S., Olyai M.R.T.B., Ali G.A.M., Sadegh H. (2019). Removal of congo red azo dye from aqueous solution by ZnO nanoparticles loaded on multiwall carbon nanotubes. Phys. E.

[76] Erdemoğlu S., Aksu S.K., Sayılkan F., İzgi B., Asiltürk M., Sayılkan H., Frimmel F., Güçer Ş. (2008). Photocatalytic degradation of Congo Red by hydrothermally synthesized nanocrystalline TiO_2_ and identification of degradation products by LC–MS. J. Hazard. Mater..

[77] Alkaykh S., Mbarek A., Ali-Shattle E.E. (2020). Photocatalytic degradation of methylene blue dye in aqueous solution by MnTiO_3_ nanoparticles under sunlight irradiation. Heliyon.

[78] Nasrollahzadeh M., Atarod M., Jaleh B., Gandomirouzbahani M. (2016). In situ green synthesis of Ag nanoparticles on graphene oxide/TiO_2_ nanocomposite and their catalytic activity for the reduction of 4-nitrophenol, congo red and methylene blue. Ceram. Int..

[79] Fowsiya J., Madhumitha G., Al-Dhabi N.A., Arasu M.V. (2016). Photocatalytic degradation of Congo red using Carissa edulis extract capped zinc oxide nanoparticles. J. Photochem. Photobiol. B Biol..

[80] Hemraj-Benny T., Tobar N., Carrero N., Sumner R., Pimentel L., Emeran G. (2018). Microwave-assisted synthesis of single-walled carbon nanotube-supported ruthenium nanoparticles for the catalytic degradation of Congo red dye. Mater. Chem. Phys..

[81] Naseem K., Farooqi Z.H., Begum R., Irfan A. (2018). Removal of Congo red dye from aqueous medium by its catalytic reduction using sodium borohydride in the presence of various inorganic nano-catalysts: A review. J. Clean. Prod..

[82] Liu J., Li J., Wei F., Zhao X., Su Y., Han X. (2019). Ag–ZnO Submicrometer Rod Arrays for High-Efficiency Photocatalytic Degradation of Congo Red and Disinfection. ACS Sustain. Chem. Eng..

[83] Nasrollahzadeh M., Issaabadi Z., Sajadi S.M. (2019). Green synthesis of Cu/Al_2_O_3_ nanoparticles as efficient and recyclable catalyst for reduction of 2,4-dinitrophenylhydrazine, Methylene blue and Congo red. Compos. Part B Eng..

[84] Raj S., Singh H., Trivedi R., Soni V. (2020). Biogenic synthesis of AgNPs employing Terminalia arjuna leaf extract and its efficacy towards catalytic degradation of organic dyes. Sci. Rep..

[85] Fairuzi A.A., Bonnia N.N., Akhir R.M., Abrani M.A., Akil H.M. (2018). Degradation of methylene blue using silver nanoparticles synthesized fromimperata cylindricaaqueous extract. IOP Conf. Ser. Earth Environ. Sci..

[86] Allen J.P., Scanlon D.O., Watson G.W. (2011). Electronic structures of silver oxides. Phys. Rev. B.

[87] Gouveia A.F., Sczancoski J.C., Ferrer M.M., Lima A.S., Santos M.R.M.C., Li M.S., Santos R.S., Longo E., Cavalcante L.S. (2014). Experimental and Theoretical Investigations of Electronic Structure and Photoluminescence Properties of β-Ag_2_MoO_4_ Microcrystals. Inorg. Chem..

[88] Bonnia N.N., Kamaruddin M.S., Nawawi M.H., Ratim S., Azlina H.N., Ali E.S. (2016). Green Biosynthesis of Silver Nanoparticles Using ‘Polygonum Hydropiper’ and Study its Catalytic Degradation of Methylene Blue. Procedia Chem..

[89] Bouafia A., Laouini S.E., Ouahrani M.R. (2020). A Review on Green Synthesis of CuO Nanoparticles using Plant Extract and Evaluation of Antimicrobial Activity. Asian J. Res. Chem..

[90] Bouafia A., Laouini S.E. (2020). Plant-Mediated Synthesis of Iron Oxide Nanoparticles and Evaluation of the Antimicrobial Activity: A Review. Mini-Rev. Org. Chem..

[91] Abdullah J.A.A., Salah Eddine L., Abderrhmane B., Alonso-González M., Guerrero A., Romero A. (2020). Green synthesis and characterization of iron oxide nanoparticles by pheonix dactylifera leaf extract and evaluation of their antioxidant activity. Sustain. Chem. Pharm..

[92] Bouafia A., Laouini S.E. (2020). Green synthesis of iron oxide nanoparticles by aqueous leaves extract of Mentha Pulegium L.: Effect of ferric chloride concentration on the type of product. Mater. Lett..

[93] Bouafia A., Laouini S.E., Tedjani M.L., Ali G.A.M., Barhoum A. (2021). Green biosynthesis and physicochemical characterization of Fe_3_O_4_ nanoparticles using Punica granatum L. fruit peel extract for optoelectronic applications. Text. Res. J..

